# Author Correction: Thickness-dependent magnetic order in CrI_3_ single crystals

**DOI:** 10.1038/s41598-020-58155-8

**Published:** 2020-01-24

**Authors:** Yu Liu, Lijun Wu, Xiao Tong, Jun Li, Jing Tao, Yimei Zhu, C. Petrovic

**Affiliations:** 1 Condensed Matter Physics and Materials Science Department, Brookhaven National Laboratory, Upton, New York, 11973 USA; 2 Center for Functional Nanomaterials, Brookhaven National Laboratory, Upton, New York, 11973 USA

Correction to: *Scientific Reports* 10.1038/s41598-019-50000-x, published online 19 September 2019

The Acknowledgements section in this Article is incomplete.

“Work at Brookhaven National Laboratory was supported by US DOE, Office of Science, Office of Basic Energy Sciences under contract DE-SC0012704.”

should read:

“This work was funded by the Computation Material Science Program (Y.L. and C.P.). TEM studies at Brookhaven National Laboratory were supported by US DOE, Office of Science, Office of Basic Energy Sciences under contract DE-SC0012704.”

